# Tubercular infection in children living with adults receiving Directly Observed Treatment Short Course (DOTS): a follow up study

**DOI:** 10.1186/s12879-020-05449-x

**Published:** 2020-10-01

**Authors:** Geetika Srivastava, M. M. A. Faridi, Shiv Sagar Gupta

**Affiliations:** 1 Department of Pediatrics, Era’s Lucknow Medical College, Era University, Sarfarazganj, Hardoi Road, Lucknow, Uttar Pradesh 226003 India; 2 Neera Hospital, Mahanagar Extension, Lucknow, Uttar Pradesh 226006 India

**Keywords:** Adult TB, Child contacts, Contact tracing, Follow-up

## Abstract

**Background:**

Children living with sputum smear-positive adult tuberculosis (TB) patients are vulnerable to acquire tubercular infection. Contact tracing is an important strategy to control tubercular infection in the community. This study was done to find out prevalence of tuberculosis and tubercular infection in children living with sputum smear-positive adult patients receiving DOTS at recruitment and to find out incidence of tubercular infection and disease in these children on follow up.

**Method:**

Children (< 15 years) living in contact with adults on DOTS were grouped as < 6 years and 6–14 years. They were further sub grouped as being - uninfected, infected, diseased and on prophylaxis and were followed at 3, 6 and 9 months. Tuberculin skin test (TST) and chest X-ray were done.

**Results:**

At recruitment 152 children were enrolled and 21.1% (*n* = 32) had TB. On follow up, 4.3% (*n* = 5), 5.8% (*n* = 6) and 11.6% (*n* = 11) children developed TB after 3, 6 and 9 months respectively.9 children did not come for the last follow up so the overall prevalence of TB disease at 9 months was 37.7% (*n* = 54). Out of the 128 children with TST reading 23.4% (*n* = 30) child contacts were found to be infected already at recruitment. The incidence of TST conversion was 20.7% (*n* = 18), 26.9% (*n* = 18) and 16.3% (*n* = 7) respectively. The overall prevalence of tubercular infection in the children, who were in contact with TB patients for 9 months was 74.5% (*n* = 73).

**Conclusion:**

About half the children were either suffering from TB or tubercular infection on recruitment. During 9 months follow up 22 unaffected children developed disease and 43acquired infection.

## Background

TB is a leading cause of mortality and morbidity in children. World Health Organization (WHO) TB statistics for 2019 estimated 10.0 million cases of TB globally of which about 10% (1 million) were less than 15 years of age [1]. Most of the children acquire tubercular infection from the sputum smear positive adults, either parents or siblings, living in the family [2]. Contact survey is a procedure for identifying children exposed to adult patient with infectious TB living with them. It is an important tool for active case finding [3]. A systematic review and meta-analysis on contact survey, which included Indian studies as well, has reported that up to 34.7% children living with adults suffering from TB were already infected with the tubercular infection [4]. Similarly in a reverse contact survey, it was found that 15.7% adults were suffering from TB when children living with them were diagnosed with TB [5]. Some studies have reported that first-degree relatives also form an important cohort for active case finding [6].

Active case finding, prompt institution of antitubercular therapy, efficient follow up for treatment compliance and prevention of further transmission of the infection are important measures to control TB. India launched Revised National Tuberculosis Program (RNTCP) in 1993 and DOTS became an integral part of it [7]. The World Health Assembly in 2014 adopted WHO’s post-2015 End TB Strategy. Preventive treatment of the individuals of high-risk group is a key component of this strategy. Children in contact with adult TB patients form an important pool for active case finding. National Strategic Plan of India for Tuberculosis Elimination 2017–2025, mandates that all household contacts of an index adult case of TB should be screened taking a Chest X-ray [8]. Indian Academy of Pediatrics (IAP) and RNTCP guidelines recommend antitubercular prophylaxis to children less than 6 years of age in contact with a sputum smear positive patient [9]. However, no definite guidelines are there to follow child contacts who are not infected at the time of contact survey and those who are more than 5 years of age. There is not enough data on the fate of such child contacts that are found infected or uninfected, on follow up as the disease progression is faster in infected children generally within 3 months after being infected with *Mycobacterium tuberculosis*.

This study was, therefore, done to find out prevalence at recruitment of TB disease and tubercular infection in children living with sputum smear-positive adult patients, receiving DOTS therapy and to find out incidence of tubercular infection and disease in these children on follow up at 3, 6 and 9 months.

## Method

This was a prospective study conducted in the Department of Pediatrics and Department of Pulmonary Medicine in a tertiary care teaching hospital after obtaining approval from the Institutional Ethics Committee. During this period, all sputum smear-positive adult patients of TB (index cases), taking regular intensive phase treatment from two DOTS Centers, situated in the hospital and Rural Health Training Centre of the institution were contacted. Those who gave consent for their children less than 15 years of age (child contact) to participate in the study were enrolled. There were 59 adult patients of TB. Child Contacts were defined as children (< 15 years age) of both genders who were residing with the adult TB patients. Demographic details of the adult index case like age, sex, socioeconomic status using modified Kuppuswamy’s socio economic scale, patient type – new patient, transfer in from other DOTS center, treatment after default, relapse or treatment failure and category of treatment whether Category I and II under DOTS, were recorded on a proforma [7, 10, 11]. They were requested to bring their children to the DOTS center on one of the visiting days for work up.

Detailed history of each child was elicited including immunization, constitutional symptoms such as fever, cough, poor appetite, weight loss, glandular swelling in any part of the body and previous history of infectious diseases (measles and pertussis). Clinical features suggestive of extra pulmonary TB like abdominal symptoms, distension, doughy abdomen, altered bowel habits, ascites, headache, vomiting, seizures etc. were recorded. Complete general and systematic examination of the child was done including weight and BMI for age assessment using WHO growth charts [12]. Presence of BCG scar was noted. Tuberculin skin testing (TST) was performed by intradermal injection and was read 48 to 72 h later [13].An induration of 10 mm or more was taken as positive irrespective of prior BCG vaccination [9, 11]. X-ray chest of all children was taken and additional investigations such as Fine needle aspiration cytology (FNAC)/ Biopsy of lymphnodes, sputum/gastric aspirate for Acid Fast Bacilli (AFB) and ultrasonography abdomen were done if required.

Child contacts were grouped in two based on age < 6 years and 6–14 years. Again on the basis of signs and symptoms, tuberculin reaction, chest X-Ray features and other relevant investigations the children were divided in to 6 sub groups:

1. Sub Group-I: Children below 6 years of age who were asymptomatic, TST negative, having normal chest X-ray i.e. Free from tubercular infection and disease.
2. Sub Group-II: Children aged 6–14 years who were asymptomatic, TST negative, with normal chest X-ray i.e. Free from tubercular infection and disease.
3. Sub Group-III: Children less than 6 years of age who were asymptomatic, TST positive, having normal chest X-ray i.e. Tubercular infection, no disease.
4. Sub Group-IV: Children in the age group of 6–14 years who were asymptomatic, TST positive with normal chest X-ray i.e. Tubercular infection, no disease.
5. Sub Group V: Children below 6 years of age found to be having tubercular disease i.e. clinical signs, symptoms and chest X-ray suggestive of TB.
6. Sub Group VI: Children 6–14 years of age having tubercular disease i.e. having clinical signs, symptoms and chest X-ray suggestive of TB.

All children from Sub Group-I to Sub Group-IV were followed up at 3 (+ 2 weeks), 6 (+ 2 weeks) and 9 (+ 2 weeks) months for evidence of tubercular infection by TST or for development of tubercular disease.

Children in Sub Group I and III were fulfilling the criteria of receiving isoniazid prophylaxis for 6 months and hence were started on it [7, 9]. Children in Sub Group-V and Sub Group-VI were suffering from TB. They were put on antituberculosis treatment under DOTS program and were excluded from the follow ups. Children, in whom TST was administered but could not be read because they did not turn up for the interpretation, were considered uninfected (TST negative) at that visit and were kept in follow up. Repeat TST was performed on subsequent follow up visit for such children. Data of such children was included for the disease prevalence as signs symptoms and chest X ray was available, but for calculating the prevalence of infection at that particular visit they were excluded.

At each follow-up detailed history of the constitutional symptoms and complete physical examination including anthropometry of the child was done. If child exhibited symptoms suggestive of TB; chest X-ray was done. If tuberculin test was found negative it was repeated at the subsequent follow up. Considering the booster phenomena, an increment in TST size of more than 6 mm from the previous reading was taken as positive [14]. If a child became TST positive on a follow up visit after being TST negative in the previous visit, it was reported as TST conversion.

### Statistical analysis

The characteristics of the variables were described using frequency (n) and percentage (%) for categorical data and using the mean and standard deviation (SD) for continuous data. Prevalence of TB infection in the children was calculated as the number of TST positive cases divided by the total number of children with TST reading. Prevalence of TB disease was calculated by dividing number of diseased children by total number of children.

Incidence of TB infection was calculated by dividing the number of newly diagnosed children having positive TST or TST conversion divided by total number of children having TST reading done at that particular visit. Incidence of TB disease was calculated by dividing the newly diagnosed children having TB disease by number of children present for that follow up.

## Results

A total of 59 adult sputum smear-positive pulmonary TB patients with 152 children living with them were enrolled in the study thus the ratio of child contacts to adult index patient was 2.58.

### Index case

Table 1 shows the demographic characteristics of the adult index cases. Age of the patients ranged between 18 and 65 years and 24 (41%) of patients were male. The DOTS therapy was given under category-1 to 42(71%) patients and 38 (64%) of them were new cases.

**Table 1:** Demographic profile and general characteristics of index subjects (*n* = 59) and their child contacts (*n* = 152)

**S.No.**	**Characteristic of Adult Tuberculosis Patients**	
1.	Age of index case in years $\overline{X}$ ± SD (Range)	29.12 ± 9.38 (18–65)
2.	Male: Female ratio of index case	24:35
3.	House (No. of Rooms) $\overline{X}$ ± SD (Range)	1.85 ± 1.00 (1–5)
4.	Socioeconomic status	n (%)
	Upper lower class	4 (6.8%)
	Lower middle class	4 (6.8%)
	Upper middle class	19 (32.2%)
	Upper class	32 (54.2%)
5.	Treatment Category of index case	n (%)
	I	42 (71.2%)
	II	17 (28.8%)
6.	Patient Type (index case)	n (%)
	New	38 (64.4%)
	Transfer in	5 (8.5%)
	Treatment after Default	12 (20.3%)
	Relapse	4 (6.8%)
	Failure	0
7.	Sputum for AFB of index case at Recruitment	n (%)
	Scanty	11 (18.6%)
	1+	11 (18.6%)
	2+	14 (23.7%)
	3+	23 (39.0%)
	**Characteristic of Child Contacts**	*N* = 152
8.	Age of child contacts $\overline{X}$ ± SD (Range) in years	6.88 ± 3.89 (0.1–13)
9.	Male: Female ratio of child contacts	78:74
10.	BCG Scar	n (%)
	Yes	102 (67.1%)
	No	50 (32.9%)
11.	Nutrition Status of child contacts	
	Normal nutrition	97 (63.8%)
	Moderate malnutrition	52 (34.2%)
	Severe malnutrition	3 (1.9%)

### Child contacts

The age of the child contacts ranged from 1 month to 13 years comprising of 55 children and 97 children < 6 years and ≥ 6 years respectively. There were 78(51%) boys. BCG scar was seen in 102 (67%) children. The weight and BMI for age was within normal limits in 97 (64%) children according to WHO criteria. Constitutional symptoms (65.6%) and cough (62.5%) were the prominent findings in the children suffering from TB (Table 1).

**Fig. 1:**
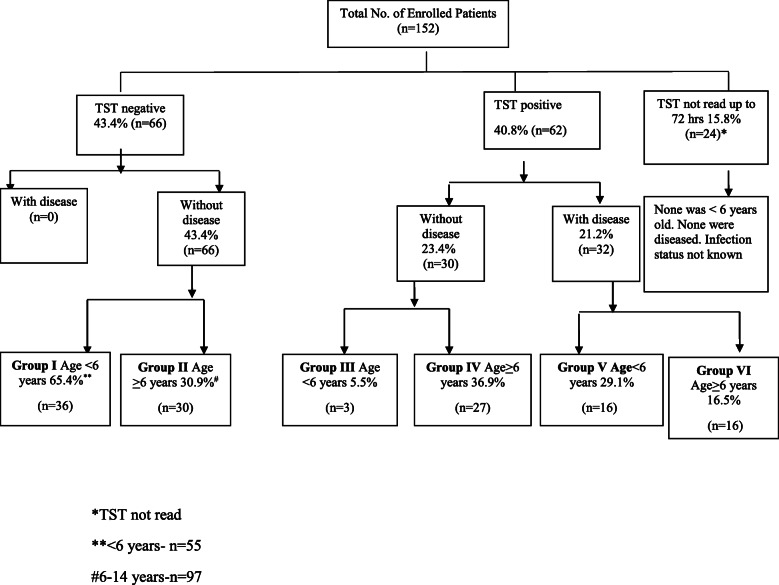
Tubercular status of child contacts at recruitment

PPD was injected to 152 child contacts at recruitment but TST could not be read in 24 children as they either reported late, beyond 72 h or did not turn up; none of them were less than 6 years old and none had any symptoms or chest x-ray suggestive of tubercular disease. Out of the remaining 128 children, 36 children were included in group-1, 30 children belonged to group-II, 3 child contacts were in group-III, group-IV comprised of 27 children and group V and VI had 16 children each. (Fig. 1) A total of 39 patients belonging to Subgroup I and III were put on isoniazid prophylaxis.

The eligible child contacts for TST were 90, 67, and 43 at 3 months, 6 months and 9 months respectively. Three children < 6 years old did not report for TST at first follow up.

The overall prevalence of tubercular infection in child contacts at recruitment was 23.4% (*n* = 30). The prevalence of tubercular infection was 7 times higher in children 6–14 years of age compared to child contacts < 6 years old (36.9% vs 5.5%) (Table 2). Further only one child contact was infected over 9 months follow up among children aged < 6 years. On the other hand children aged 6–14 years continued to acquire tubercular infection and 33.3% (*n* = 18), 58.1% (*n* = 18) and 85.7% (*n* = 6) child contacts were found infected on quarterly assessment till 9 months respectively (Table 2).

**Table 2:** Acquisition of tubercular infection (TST + ve) in children living with household adult TB patients

Visit	TB Infection (TST + ve) n (%)			Children for TST
	Age < 6 yrs	Age 6–14 yrs	Total	
Recruitment	3 (5.5)	27 (36.9)	30 (23.4)	
	*N* = 55	*N* = 73^a^	*N* = 128	
3 Months	0	18 (33.3)	18 (20.7)	90
	*N* = 33^b^	*N* = 54	*N* = 87	
6 Months	0	18 (58.1)	18 (26.9)	67
	*N* = 36	*N* = 31	*N* = 67	
9 Months	1 (2.8)	6 (85.7)	7 (16.3)	43
	*N* = 36	*N* = 7	*N* = 43	

^a^TST not read in 24 children

^b^3 children did not report

TB disease was diagnosed in 21.1% (*n* = 32/152) child contacts living with the adult TB patients at the time of recruitment. Interestingly the prevalence of TB was inversely proportional to the age, with the prevalence in child contacts < 6 years being almost double that of older children (29.1% vs 16.5%). Out of 55 child contacts aged < 6 years 39 children were eligible for evaluation of the TB disease on follow up; 92.3% (*n* = 36) children reported at 3 month, 6 months and 9 months follow. Among them only one child (2.7%) developed TB on follow up. On the other hand, among 6–14 years old child contacts 8 and 6 children did not report at 6 months and 9 months for evaluation of TB (Table 3). In these children 6.2% (*n* = 5), 8.8% (*n* = 6), and 16.9% (*n* = 10) children developed TB at 3 monthly intervals. A total of 22 new cases of TB emerged in the child contacts during the period of 9 months. Thus 54 (37.7%) children out of 143 child contacts (9 children finally lost to at 9 months) suffered from tubercular disease over a period of 9 months from recruitment (Table 3). Among diseased children (*n* = 54) 29 children had pulmonary TB, 23 children had cervical lymphadenopathy and one child each suffered from tubercular meningitis (< 6 years) and abdominal TB.

**Table 3:** Development of tuberculosis disease over a period of 9 months in children living with household adult TB patients

Visit	TB Disease n (%)			Children to be followed
	< 6 yrs	6–14 yrs	Overall	
Recruitment	16 (29.1)	16 (16.5)	32 (21.1)	
	*N* = 55	*N* = 97	*N* = 152	
3 Months	0	5 (6.2)	5 (4.3)	120
	*N* = 36^a^	81	117	
6 Months	0	6 (8.8)	6 (5.8)	115
	*N* = 36^a^	*N* = 68^b^	*N* = 104	
9 Months	1 (2.7)	10 (16.9)	11 (11.6)	104
	*N* = 36^a^	*N* = 59^c^	*N* = 95	

^a^3 did not report

^b^8 children did not report

^c^6 children did not report

Thus, out of 152 child contacts a total of 54 children were found to have TB over a period of 9 months from the time of recruitment. In remaining 98 child contacts 73 children (74.5%) were found infected during the same period (Table 2).

## Discussion

This study was done on 152 children, < 15 years of age, and living in contact with 59 sputum smear-positive pulmonary adult TB patients who were receiving DOTS therapy. Children were evaluated for the development of tubercular infection and TB disease over a period of 9 months; more than 96% children reported for follow up. The study showed that 21.1% (*n* = 32) children were suffering from TB at the time of contact survey and the number rose to 37.7% (*n* = 54) at 9 months. During the same time 23.4% (*n* = 30) and 74.5% (*n* = 73) children were detected to have tubercular infection on the basis of positive TST.

WHO defines contacts as those residing in the same household for one or more nights or in frequent contact over an extended time period during 3 months before start of the current treatment in the adult patient [15]. Household contacts constitute an important cohort for case finding and implementation of screening of these contacts can prevent more than 100,000 cases of TB and almost 100,000 deaths in children less than 15 years [16].

Our study revealed that almost 1/5th of all child contact surveyed were already suffering from TB and that prevalence was higher in younger children. Almost equal number of children were suffering from tubercular infection at recruitment. The prevalence of TB in children (< 15 years) living in contact with household index case ranges from 4.8–16.9% [17–20]. Various studies report prevalence of tubercular infection in child contacts ranging from 24.4–69.2% [21]. Our study showed that prevalence of TB at recruitment was 1.76 times higher (29.1%) in children aged < 6 years compared to children between 6 and 14 years (16.5%). The higher prevalence of TB in young children could be due to the fact that they have an impaired immune response thereby facilitating progression of TB infection to disease [6]. TB being a droplet infection spreads easily in close contacts specially from female index cases as they are the primary caregivers for children [22]. The transmission of TB depends on many epidemiological factors like time spent with the index case, proximity, the living conditions of the household, the number of rooms etc. [5] BCG vaccination has a proven protective role [23]. Age ≤ 5 years is an important risk factor for developing the disease which is established in our study too [24].

There were 22 new cases of TB diagnosed during 9 months follow up. Out of them only one patient was less than 6 years of age and was receiving isoniazid prophylaxis. Isoniazid prophylaxis has a proven role in the prevention of TB in child contacts of sputum smear-positive patient. Many studies done have proved good outcome of prophylaxis based on adherence to it [25, 26]. Despite the proven benefits of prophylaxis it is not being routinely given especially in developing countries. A study from India reports that only 33% child contacts are started on isoniazid prophylaxis [27].

Seeing the significant proportion of children 6–14 years of age developing TB and tubercular infection in our study, it is prudent to suggest that all children and adolescent contacts should be kept under close surveillance, and isoniazid prophylaxis to be given to them. World Health Organization has now recommended preventive treatment for children less than 15 years in high incidence countries [15].

Family physicians and primary health care workers must immunize all children including BCG on time. Pediatricians and family physicians must counsel family members suffering from TB for taking precautionary measures like cough etiquettes, using mask, frequent hand washing and avoiding proximity with the children and to take steps for building nutrition, vaccination against measles and pertussis and isoniazid prophylaxis to eligible contacts [28]. The observations of this study will be very useful in high TB burden countries in controlling the disease in the community. Isoniazid prophylaxis to all child contacts < 15 years of age and early detection of the disease in both child and adult contacts will be an important strategy for managing the scourge of TB.

### Limitations

Sputum/gastric aspirate for Acid fast bacilli (AFB) was not taken in all children due to logistic constraints. We did not correlate epidemiological factors such as socio-economic status, nutritional factors and gender of the adult patients with the prevalence of the disease and tubercular infection in the child contacts. There were 24 children whom TST could not be read at recruitment, although they did report for follow up at later visits, the exact prevalence of tubercular infection at recruitment could be an underestimate. At the time when this study was carried out Gene Expert/ Cartridge based nucleic acid amplification test, CBNAAT, was not available in the public domain so it was not carried out.

## Conclusion

Our study confirms that large number of children (21.1%) living with adult sputum smear-positive TB patients suffer from active disease and almost equal number of child contacts (23.4%) harbor latent infection of TB. During 9 months follow up the children continue to get exposed and prevalence of active disease and TB infection rise to 37.7 and 74.5%respectively.

## Data Availability

The datasets used and/or analyzed during the current study are available from the corresponding author on reasonable request.

## Abbreviations

DOTS: Directly Observed Treatment Short Course
WHO: World Health Organization
TB: Tuberculosis
IAP: Indian Academy of Pediatrics
RNTCP: Revised National Tuberculosis Control Program
TST: Tuberculin skin test
PPD: Purified protein derivative
BCG: Bacillus calmette guerin vaccine
TU: Tuberculin units
LTBI: Latent tubercular infection
CBNAAT: Cartridge based nucleic acid amplification test
